# Three Minimum Variance Distortionless Response Algorithms Based on Quaternion Model for Single MEMS Vector Hydrophone

**DOI:** 10.3390/mi17101137

**Published:** 2026-09-29

**Authors:** Shan Zhu, Yanan Geng, Keqiang Mu, Yongwei Li, Feng Li, Zhiqiang Gao, Yunyue Zhang, Zhicheng Zhao

**Affiliations:** 1 Department of Automation, Taiyuan Institute of Technology, Taiyuan 030008, China; liyw@tit.edu.cn (Y.L.); lifeng@tit.edu.cn (F.L.); gaozhiqiang@tit.edu.cn (Z.G.); zhangyunyue@tit.edu.cn (Y.Z.); 1996049@tyust.edu.cn (Z.Z.); 2 State key Laboratory of Extreme Environment Optoelectronic Dynamic Measurement Technology and Instrument, North University of China, Taiyuan 030051, China; gynblue@163.com; 3 North Automatic Control Technology Institute, Taiyuan 030006, China; mkq042896@163.com

**Keywords:** single MEMS vector hydrophone, quaternion, MVDR, direction-of-arrival estimation

## Abstract

To address the limitations of the traditional long-vector model, which cannot adequately preserve the inherent orthogonal structure among channels in the signal processing of a single vector hydrophone, three minimum variance distortionless response (MVDR) direction-of-arrival estimation algorithms based on the quaternion model are proposed for a single MEMS vector hydrophone. Compared with the traditional long-vector model, the quaternion can retain the structural relationship between channels in a more compact data representation form. First, based on the output characteristics of the sound pressure and dual-channel vibration velocity of the two-dimensional MEMS vector hydrophone, three quaternion signal models—namely Q-VV, Q-PVV, and Q-PV—are constructed. Subsequently, the corresponding MVDR spatial spectra are derived in the quaternion domain. To enhance the direction-of-arrival resolution performance under low-signal-to-noise-ratio (SNR) conditions, the algorithms are then improved by incorporating subspace decomposition theory. The simulation results show that the three proposed quaternion-based MVDR algorithms can all realize effective direction-of-arrival estimation for a single target. When the signal-to-noise ratio is greater than −4 dB, the direction estimation error is less than 1°. The results of dynamic bearing trajectory tests and fixed-point direction-of-arrival estimation experiments indicate that the proposed method can realize the continuous and stable tracking of the target azimuth. Among the involved algorithms, Q-PV-MVDR can effectively suppress false targets, with an average relative angle orientation error of 0.5°.

## 1. Introduction

Underwater target bearing estimation is a key link in underwater acoustic detection, target tracking, active and passive positioning and other tasks [1,2,3,4]. Different from the traditional scalar hydrophone, which can only acquire sound pressure information, the vector hydrophone can simultaneously measure sound pressure and particle velocity. It utilizes the frequency-independent dipole directivity of the particle velocity channel to extract directional information about the sound field, thus exhibiting obvious advantages in detecting low-frequency weak targets [5]. Among them, the micro-electro-mechanical system (MEMS) vector hydrophone has the characteristics of a small size, low power consumption, and easy integration, and it can be used for the common point measurement of sound pressure and vibration velocity [6,7,8,9]. A single sensor can simultaneously obtain scalar information and vector information about the sound field. At the same time, the development of MEMS processing, sensitive material preparation and device calibration technology also provides important support for its structural optimization and performance improvement. Shi et al. designed an integrated thermoelectric parameter test chip to realize multi-parameter synchronous measurement, which provides a new device design idea for the calibration of the electrical and thermal properties of MEMS sensitive films [10]. In terms of sensitive materials and microstructure design, Ahangari et al. systematically summarized layered metal oxide electrode materials, which provided a reference for the selection and performance optimization of MEMS sensitive materials [11]. Hsu et al. realized the controllable driving of microcavity fluid by using a surface acoustic wave, which provides a reference for the design of acoustic–fluid coupling in microstructures [12]. Ganguly et al. summarized the application of magnetic polymer microtubules in biosensors, and their flexible microstructure preparation method can also provide inspiration for the design and processing of vector hydrophone sensitive components [13]. The above technical progress expands the technical path of MEMS vector hydrophones in structural design, material selection, performance calibration and error compensation.

In order to improve the performance of azimuth estimation under complex noise conditions, subsequent studies have begun to use acoustic energy flow, signal reconstruction and matched filtering to enhance target features. Zhang et al. used a single MEMS vector hydrophone to detect acoustic energy flow, used the energy center of the acoustic energy distribution as the azimuth estimation result, and obtained a directional error of 2.21° under the condition of a 0 dB signal-to-noise ratio (SNR) [14]. Aiming at the problem of the performance degradation of traditional methods under multi-source and noise interference conditions, Guo et al. proposed a vector signal reconstruction (VSR) method. The simulation results show that when the SNR is higher than −5 dB, the azimuth root mean square error of the single-frequency signal can be controlled within 2° [15]. Geng et al. further proposed an adaptive noise residual accumulation method based on the frequency domain deconvolution of sound pressure and vibration velocity based on matched filtering, which achieved an azimuth estimation error of no more than 2° in a lake test with an SNR of −7 dB [16]. In addition, Zhang et al. used Boltzmann machine learning to extract and compensate array device deviations, providing new ideas for channel calibration and the error suppression of vector sensors [17]. The above research shows that azimuth estimation performance under low-SNR conditions can be improved by signal enhancement, structure reconstruction and error compensation, but the data model usually still adopts the traditional real or complex long-vector form.

Early research on high-resolution DOA estimation based on acoustic vector sensors was mainly based on the Capon/MVDR beamforming framework. Hawkes et al. systematically studied the beamforming and Capon direction estimation methods of acoustic vector sensors and proved that higher spatial resolution can be obtained by using joint information about sound pressure and particle velocity [18]. In the traditional long-vector model, sound pressure and particle velocity components along different directions are sequentially arranged to construct a multidimensional vector, based on which covariance matrices and spatial spectral functions are established. This representation boasts a simple structure and facilitates the direct implementation of classical array signal processing approaches including beamforming, Multiple signal classification (MUSIC) and minimum variance distortionless response (MVDR). However, the long-vector model merely performs the algebraic concatenation of different output channels. It cannot explicitly describe the spatial orthogonality among particle velocity components, making it difficult to preserve the integrity of multi-component signals from a single vector hydrophone during data operations. Especially under low-SNR conditions, the covariance matrix in the traditional model is vulnerable to finite snapshots, background noise and channel errors, resulting in widened spatial spectrum peaks, increased spurious peaks and degraded azimuth resolution.

With the development of acoustic vector sensor research, how to make full use of multi-channel vector information and maintain the internal coupling relationship between different physical quantities has become a new research direction. It has been pointed out that the advanced signal representation method and multidimensional information processing technology oriented to the characteristics of vector sensors are important development directions for improving the performance of AVSs [19]. Quaternions have a real part and three mutually orthogonal imaginary parts, which can describe multi-channel vector information under the unified algebraic framework and provide an effective tool for maintaining the coupling relationship and orthogonal structure between different channels. Existing quaternion DOA estimation research mainly focuses on electromagnetic vector sensors and acoustic vector sensor arrays. Wang et al. discussed the quaternion direction estimation problem of two-dimensional vector sensors under Gaussian noise conditions [20]. Gong et al. proposed an electromagnetic vector sensor array DOA estimation method based on quaternion algebra. The simulation results show that when the SNR is higher than −6 dB, the azimuth error can be less than 1° [21]. Meng et al. constructed a quaternion conjugate enhanced output model for non-circular signals, which improved the accuracy of azimuth estimation while reducing computational complexity [22]. Wang et al. constructed a quaternion model using different velocity components of acoustic vector sensors and realized azimuth ambiguity resolution based on the rotation relationship between subarray quaternion steering vectors [23]. Sadegh et al. applied the quaternion polynomial rooting method to a two-component electromagnetic vector sensor array, which improved the resolution of close-range targets under low-SNR conditions while reducing the amount of calculation [24]. Lou et al. further analyzed the theoretical performance of the quaternion MUSIC algorithm from the aspects of noise subspace disturbance and estimation error statistical characteristics [25]. Nevertheless, their work lacks analyses based on simulation and experimental data. Moreover, the MUSIC algorithm requires prior knowledge of the source number, imposes heavy computational burden, and suffers limitations when dealing with coherent sources.

Previous studies have shown that the quaternion method has advantages in multi-channel vector information expression, orthogonal structure preservation and subspace parameter estimation, but related research is mainly on multi-element vector sensor arrays. A single MEMS vector hydrophone can obtain sound pressure and two orthogonal particle velocity components at the same point, which can be regarded as a vector sensing system with zero array element spacing. However, the mapping method of its three signals in quaternion space and its influence on MVDR performance still lack systematic research. Therefore, this paper focuses on the DOA estimation of a single MEMS vector hydrophone. Three quaternion signal models are constructed by combining the quaternion modeling method with the MVDR algorithm, and the influence of the noise component and limited snapshot error on spatial spectrum estimation is reduced by subspace decomposition. Finally, MATLAB R2021b simulation and low-frequency test system experiments are used to verify the proposed method, focusing on evaluating its azimuth resolution and estimation robustness under low-SNR conditions. It should be pointed out that there are still some differences between the controlled experimental environment and the real ocean acoustic scene. Factors such as multipath propagation, environmental disturbance and the sound velocity profile change in the actual shallow water environment will further increase the uncertainty of underwater acoustic propagation [26]. In future work, the adaptability of the proposed method under complex propagation conditions will be verified by combining the actual shallow sea environment data and sea test conditions.

## 2. MVDR Algorithm Based on Quaternion Model

### 2.1. Definition of Quaternions

Quaternions were proposed by Hamilton in 1843 as a kind of hypercomplex number with one real part and multiple imaginary parts. They can be regarded as an extension of complex numbers from the two-dimensional plane to the four-dimensional space and can be expressed in the following form [27]:

*q* = *a* + *ib* + *jc* + *kd* (1)

where *a*, *b*, *c*, *d* are real numbers, and *i*,*j*,*k* denote the imaginary units of quaternions, which satisfy the following conditions: *i*^2^ = *j*^2^ = *k*^2^ = *ijk* = −1, *ij* = −*ji* = *k*, *jk* = −*kj* = *i*, and *ki* = −*ik* = *j*. *a* is defined as the real part of the quaternion. When *a* = 0, *q* is referred to as a pure quaternion.

For the convenience of processing, quaternions are usually written in the following form:

*q* = *α* + *βj* = (*a* + *ib*) + (*c* + *id*)*j* (2)

### 2.2. Quaternion Model of MEMS Vector Hydrophone

The MEMS vector hydrophone is composed of a sound pressure channel and two velocity channels. It outputs sound pressure and orthogonal two-dimensional velocity components at the same point. Under the assumption of a far-field plane wave, the wave impedance of the velocity component takes a constant of 1, and the received signal model of the MEMS vector hydrophone is as follows:

(3) $$\left\{\begin{array}{l} p(t)=s(t)+n_{p}(t)\\ v_{x}(t)=s(t)\cos\theta +n_{x}(t)\\ v_{y}(t)=s(t)\sin\theta +n_{y}(t) \end{array}\right.$$

where *θ* represents the azimuth angle between the target signal and the *x*-axis, with *θ* ∈ (0, 2*π*)*. n_p_*(*t*), *n_x_*(*t*), *n_y_*(*t*) represents noise, which is not related to the sound source emission signal *s*(*t*), and different noise components are independent of each other.

Without considering the influence of noise, if *p*(*t*), *v_x_*(*t*), *v_y_*(*t*) is represented by a column vector, the output of a single MEMS vector hydrophone can be expressed as follows [28]:

(4) $$z(t)={\left[p(t)\;v_{x}(t)\;v_{y}(t)\right]}^{T}=s(t){\left[1\;\cos\theta \;\sin\theta \right]}^{T}=s(t)a(\theta )$$

where $a(\theta )={\left[1\;\cos\theta \;\sin\theta \right]}^{T}$ denotes the steering vector of the single MEMS vector hydrophone.

According to Equation (2), the following quaternion model (Q-VV method) is constructed for the vibration velocity information *v_x_* and *v_y_* about the single output of the MEMS vector:

(5) $$\begin{array}{l} q=v_{x}+jv_{y}=(r_{x}+iI_{x})+j(r_{y}+iI_{y})\\ \;\;=r_{x}+iI_{x}+jr_{y}+kI_{y} \end{array}$$

Here, sound pressure and vibration velocity are analytical signals; that is, *r_x_*, *r_y_* is the real part of the two vibration velocity components, and *I_x_*, *I_y_* is the imaginary part of the two vibration velocity components. According to the properties of quaternions, the above formula can constitute a complete quaternion.

According to Equations (3) and (5), the quaternion output model of a two-dimensional MEMS vector hydrophone is the following:

(6) $$\begin{array}{c} z(t)=s(t)\cos\theta +\mathrm{js}(t)\sin\theta \\ \;\;=s(t)(\cos\theta +j\sin\theta ) \end{array}$$

Accordingly, the quaternion steering vector can be formulated as follows:

*a_q_* = cos*θ* + *j*sin*θ* (7)

Similarly, Q-PVV and Q-PV quaternion output models are constructed for sound pressure and vibration velocity information about the single output of the MEMS vector:

(8) $$\begin{array}{l} q=(p+v_{x})+v_{y}j\\ \;\;=(r_{p}+I_{p}i)+(r_{x}+I_{x}i)+(r_{y}+I_{y}i)j\\ \;\;=(r_{p}+r_{x})+(I_{p}+I_{x})i+r_{y}j+I_{y}k \end{array}$$

(9) $$\begin{array}{l} q=(p+v_{x})+(p+v_{y})j\\ \;\;=(r_{p}+I_{p}i)+(r_{x}+I_{x}i)+(r_{p}+I_{p}i+r_{y}+I_{y}i)j\\ \;\;=(r_{p}+r_{x})+(I_{p}+I_{x})i+(r_{p}+r_{y})j+(I_{p}+I_{y})k \end{array}$$

At this point, the quaternion output model of the two-dimensional MEMS vector hydrophone is expressed as follows:

(10) $$\begin{array}{c} z(t)=s(t)+s(t)\cos\theta +\mathrm{js}(t)\sin\theta \\ =s(t)(1+\cos\theta +j\sin\theta ) \end{array}$$

(11) $$\begin{array}{l} z(t)=(s(t)+s(t)\cos\theta )+j(s(t)+s(t)\sin\theta )\\ \;\;\;=s(t)(1+\cos\theta +j+j\sin\theta )\\ \;\;\;=s(t)(1+\cos\theta +j(1+\sin\theta )) \end{array}$$

Similarly, the quaternion steering vector can be expressed as follows:

*a_q_*(*θ*) = 1 + cos*θ* + *j*sin*θ* (12)

*a_q_*(*θ*) = 1 + cos*θ* + *j*(1 + sin*θ*) (13)

### 2.3. Quaternion-Based MVDR Algorithm

On the basis of the above modeling, the MVDR criterion is introduced for beamforming. Its core idea is to minimize the noise and interference arriving from directions other than the target azimuth while keeping the signal power unchanged in the target direction. This is realized by adjusting the array weight vector, whose constraint condition is given as follows [29]:

(14) $$\left\{\begin{array}{l} \underset{w}{\min}w^{H}\mathrm{Rw}\\ w^{H}a(\theta )=c \end{array}\right.$$

where *R* is the covariance matrix of the MEMS vector hydrophone output, *w* denotes the weight vector, the *H* superscript represents the conjugate transpose, *a*(*θ*) represents the directional vector of the specified direction, and *c* is a constant.

In practice, due to the finite length of the data received by the array, the array covariance matrix is estimated by the following equation [30]:

(15) $$R=\frac{1}{L}\sum_{i=1}^{L}ZZ^{H}$$

where *L* is the number of time domain samples of all array elements, namely the snapshot number. It should be pointed out that for different quaternion modeling methods, the observation *Z* corresponds to different quaternion outputs, so its covariance matrix is estimated independently by the corresponding quaternion observation data, rather than using the same covariance matrix.

The objective function is constructed as follows [31]:

(16) $$J(w)=\frac{1}{2}w^{H}\mathrm{Rw}-\eta (w^{H}a(\theta )-c)$$

By taking the partial derivative of the objective function with respect to *w* and setting the partial derivative to zero, the following can be obtained:

(17) $$\frac{\partial J(w)}{\partial w}=\mathrm{Rw}-\mathrm{\eta a}(\theta )=0$$

*w* = *ηR*^−1^*a*(*θ*) (18)

The following can be obtained using the constraint condition:

*w^H^a*(*θ*) = *ηa*(*θ*)*^H^R*^−1^*a*(*θ*) = *c* (19)

This yields the following:

(20) $$\eta =\frac{c}{a{\left(\theta \right)}^{H}R^{-1}a(\theta )}$$

(21) $$w=c\frac{R^{-1}a(\theta )}{a{\left(\theta \right)}^{H}R^{-1}a(\theta )}$$

If *c* = 1, Equation (21) is exactly the MVDR beamformer.

The corresponding output average power is as follows:

(22) $$P_{\mathrm{LV}-\mathrm{MVDR}}=w^{H}\mathrm{Rw}=\frac{1}{a{\left(\theta \right)}^{H}R^{-1}a(\theta )}$$

Because the quaternion multiplication is noncommutative, the Hamilton quaternion matrix theory is used for derivation. Define the quaternion covariance matrix as a Hermitian matrix:

*R_q_* = *R_q_^H^* (23)

where *H* represents the quaternion conjugate transpose.

Combining Equations (7), (12), (13) and (22), the outputs of three quaternion-based MVDR algorithms are obtained.

(24) $$P_{Q-\mathrm{VV}-\mathrm{MVDR}}=\frac{1}{{\left(\cos\theta +j\sin\theta \right)}^{H}R_{q}^{-1}(\cos\theta +j\sin\theta )}$$

(25) $$P_{Q-\mathrm{PVV}-\mathrm{MVDR}}=\frac{1}{{\left[(1+\cos\theta )+j\sin\theta \right]}^{H}R_{q}^{-1}[(1+\cos\theta )+j\sin\theta ]}$$

(26) $$P_{Q-\mathrm{PV}-\mathrm{MVDR}}=\frac{1}{{\left[(1+\cos\theta )+(1+\sin\theta )j\right]}^{H}R_{q}^{-1}[(1+\cos\theta )+(1+\sin\theta )j]}$$

Since the three methods use different quaternion observation models, the corresponding covariance matrix is estimated independently by the corresponding observation data.

Although Equations (24)–(26) extend the MVDR algorithm from the complex domain to the quaternion domain, the MVDR algorithm does not belong to the super-resolution algorithm and has weak angle resolution capability. Therefore, the above algorithms are improved based on the principle of the MUSIC algorithm.

In the MUSIC algorithm, the eigenvalue decomposition of the covariance matrix is performed to obtain the following expression [32]:

(27) $$R=\sum_{i=1}^{N}\lambda_{i}e_{i}e_{i}^{H}+\sum_{j=N+1}^{M}\sigma^{2}e_{j}e_{j}^{H}=U_{s}\sum_{s}U_{s}^{H}+U_{n}\sum_{n}U_{n}^{H}$$

where *λ_i_* and *e_i_* are the N larger eigenvalues and their corresponding eigenvectors, respectively; *U_s_* = [*e*_1_,*e*_2_,…,*e_N_*] denotes the signal subspace; Σ*_s_* = *diag*(*λ*_1_,*λ*_2_,…,*λ_N_*); *σ*^2^ is the noise power; *e_j_* represents the *M* − *N* smaller eigenvectors; *U_n_* = [*e_N_*_+1_,…,*e_M_*] denotes the noise subspace; Σ*_n_* = *diag*(*σ*^2^,…,*σ*^2^).

According to the properties of the eigenvalue decomposition of the Hermitian matrix, the following relations hold:

(28) $$R^{-1}=\sum_{i=1}^{N}\frac{1}{\lambda_{i}}e_{i}e_{i}^{H}+\frac{1}{\sigma^{2}}\sum_{j=N+1}^{M}e_{j}e_{j}^{H}$$

(29) $${\left(\sigma^{2}R^{-1}\right)}^{m}={\sum_{i=1}^{N}\left(\frac{\sigma^{2}}{\lambda_{i}}\right)}^{m}e_{i}e_{i}^{H}+\sum_{j=N+1}^{M}e_{j}e_{j}^{H}$$

Obviously, $\underset{m\to \infty }{\lim}({\frac{\sigma^{2}}{\lambda_{i}})}^{m}=0,\underset{m\to \infty }{\lim}{\left(\sigma^{2}R^{-1}\right)}^{m}=\sum_{j=N+1}^{M}e_{j}{e_{j}}^{H}=U_{n}{U_{n}}^{H}$.

Therefore, Equations (24)–(26) can be rewritten as follows:

(30) $$P_{Q-\mathrm{VV}-\mathrm{MVDR}}=\frac{1}{{\left(\cos\theta +j\sin\theta \right)}^{H}R_{q}^{-m}(\cos\theta +j\sin\theta )}$$

(31) $$P_{Q-\mathrm{PVV}-\mathrm{MVDR}}=\frac{1}{{\left[(1+\cos\theta )+j\sin\theta \right]}^{H}R_{q}^{-m}[(1+\cos\theta )+j\sin\theta ]}$$

(32) $$P_{Q-\mathrm{PV}-\mathrm{MVDR}}=\frac{1}{{\left[(1+\cos\theta )+(1+\sin\theta )j\right]}^{H}R_{q}^{-m}[(1+\cos\theta )+(1+\sin\theta )j]}$$

When the value of *m* is relatively large, the direction-of-arrival estimation performance of the above formula is comparable to that of the quaternion MUSIC algorithm. In general, *m* is set to 2.

## 3. Simulation

### 3.1. Simulation 1

**Figure 1:**
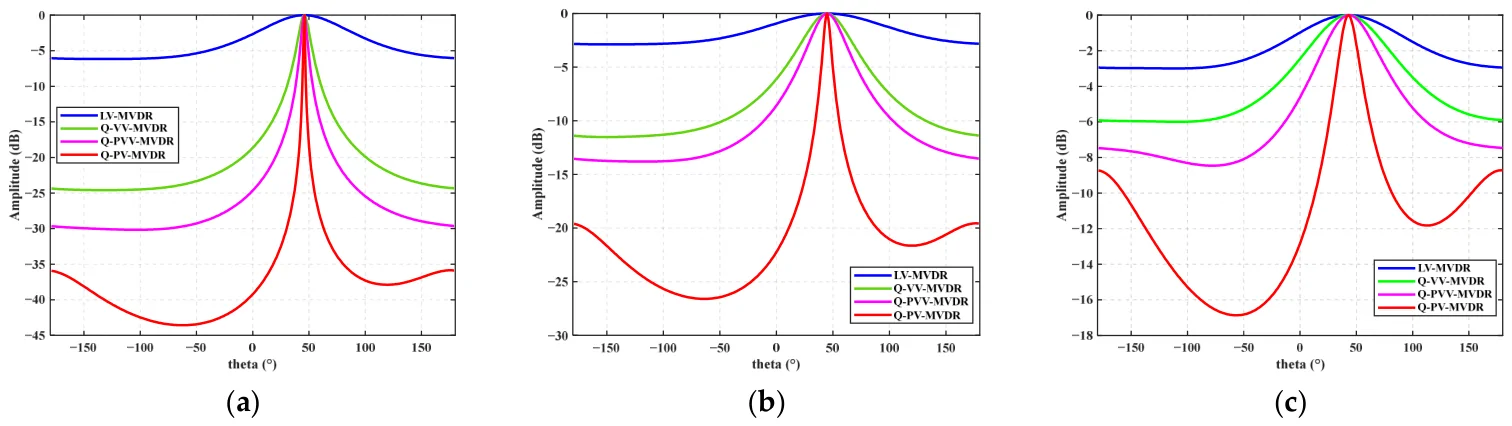
Estimation performance of MVDR algorithms for single target: (**a**) SNR = 0 dB; (**b**) SNR = −5 dB; (**c**) SNR = −10 dB.

It is assumed that a signal is incident on the two-dimensional MEMS vector hydrophone at an angle of 45°. The SNR is set to −10 dB, −5 dB and 0 dB respectively, with the snapshot number *L* = 10,000. The background noise is independent and identically distributed Gaussian white noise. Figure 1 gives the estimation results of the conventional MVDR algorithm and three quaternion MVDR algorithms based on the power correction of the inverse covariance matrix.

From the simulation results, it can be observed that under signal-to-noise ratio conditions of 0 dB, −5 dB and −10 dB, all the algorithms can realize the effective estimation of the target azimuth. Compared with the Q-VV-MVDR and Q-PVV-MVDR methods, the Q-PV-MVDR method uses the joint representation of sound pressure and vibration velocity information to provide richer signal characteristics in the quaternion observation space. At the same time, after inverse covariance matrix power correction, the spatial spectrum peak is more concentrated, and the main lobe width is further reduced, showing higher angular resolution. Therefore, the Q-PV-MVDR method achieves a more obvious peak enhancement effect under low-SNR conditions, and its estimation performance is better than that of the Q-VV-MVDR, Q-PVV-MVDR and LV-MVDR methods.

### 3.2. Simulation 2

The SNR varies from −10 dB to 10 dB. Three quaternion-based Q-MVDR algorithms and the LV-MVDR algorithm are adopted to conduct 500 independent Monte Carlo experiments at each SNR. The root mean square error (RMSE) of the DOA estimation is adopted as the evaluation criterion, and its expression is defined as follows:

(33) $$\mathrm{RMSE}=\sqrt{\frac{1}{N}E[{\left(\hat{\theta }-\theta \right)}^{2}]}$$

where $\hat{\theta }$ denotes the estimated direction of arrival of the target; *N* represents the number of independent simulation trials.

**Figure 2:**
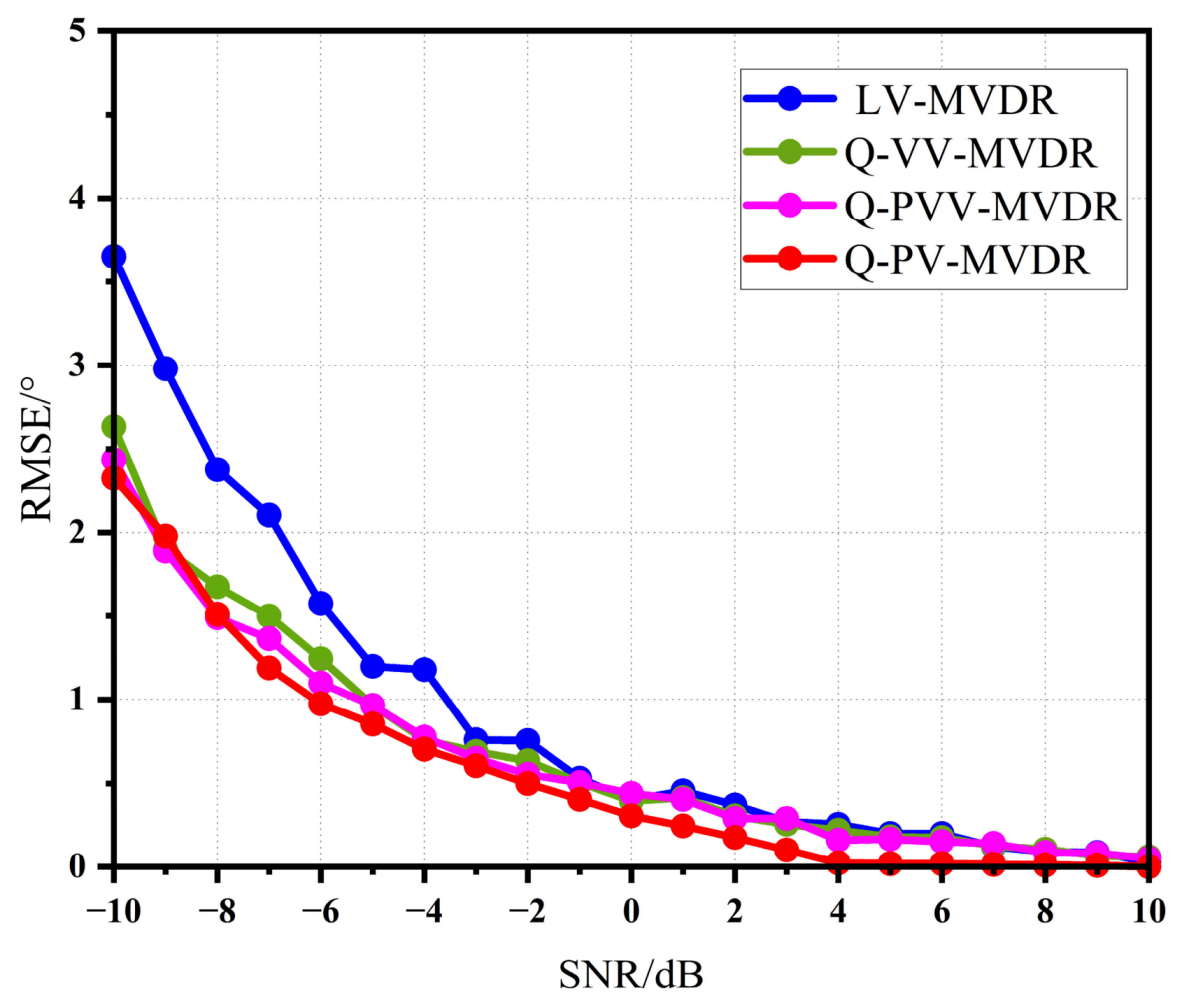
RMSE curves of MVDR algorithms for single-target estimation.

Figure 2 shows the RMSE curves of the conventional MVDR algorithm and the improved quaternion MVDR algorithms for single-target estimation. By comparing the simulation results, it can be observed that the quaternion-based MVDR algorithms achieve higher estimation accuracy for single acoustic source targets than the LV-MVDR algorithm under low-SNR conditions. The direction-of-arrival estimation accuracy ranks from high to low as Q-PV-MVDR, Q-PVV-MVDR, Q-VV-MVDR, and LV-MVDR.

### 3.3. Simulation 3

**Figure 3:**
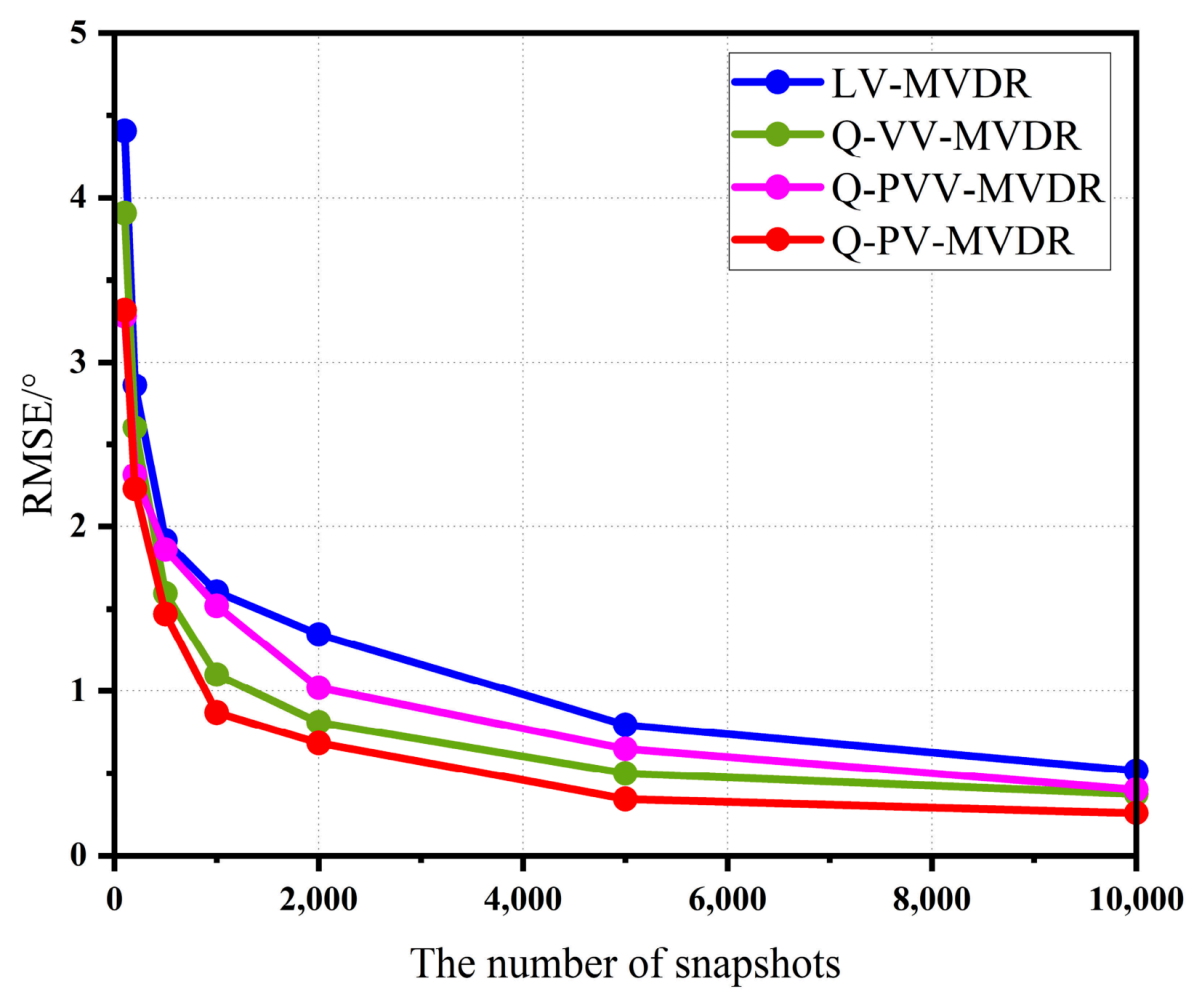
Snapshot number sensitivity simulation.

To further evaluate the robustness of the proposed algorithms under the condition of limited snapshots, a snapshot number sensitivity simulation is carried out in this section. It is assumed that a signal impinges on the two-dimensional MEMS vector hydrophone at an incident angle of 45°, and the background noise is independent and identically distributed Gaussian white noise. The SNR is fixed at 0 dB. Three quaternion-based MVDR algorithms and the LV-MVDR algorithm are employed to perform 500 independent Monte Carlo experiments for each snapshot number. The snapshot number is set within the range [100:100:10,000], and Equation (29) is adopted as the evaluation metric. The corresponding simulation results are presented in Figure 3.

The simulation results indicate that the direction-of-arrival estimation error of each algorithm gradually decreases as the number of snapshots increases. This is because a larger number of snapshots enables the sample covariance matrix to estimate the true covariance matrix more accurately, thereby concentrating the peaks of the spatial spectrum and achieving more stable target direction-of-arrival estimation. Under the condition of small snapshot numbers, the conventional method is more sensitive to the estimation error of the covariance matrix and suffers a larger angular estimation bias. In contrast, the quaternion model method can characterize the structural relationship among multi-channel outputs more compactly and thus exhibits better robustness under limited snapshots. As the number of snapshots further increases and satisfies *L* ≥ 2000, the performance of each algorithm gradually tends to be stable. Among all methods, Q-PV-MVDR achieves the minimum estimation error, which demonstrates its superior robustness under both small-sample and low-SNR conditions.

## 4. Experiment

To evaluate and verify the performance of the improved algorithm, bearing history estimation experiments and fixed-point bearing estimation experiments are carried out in a low-frequency calibration system.

### 4.1. Bearing History Experiment

**Figure 4:**
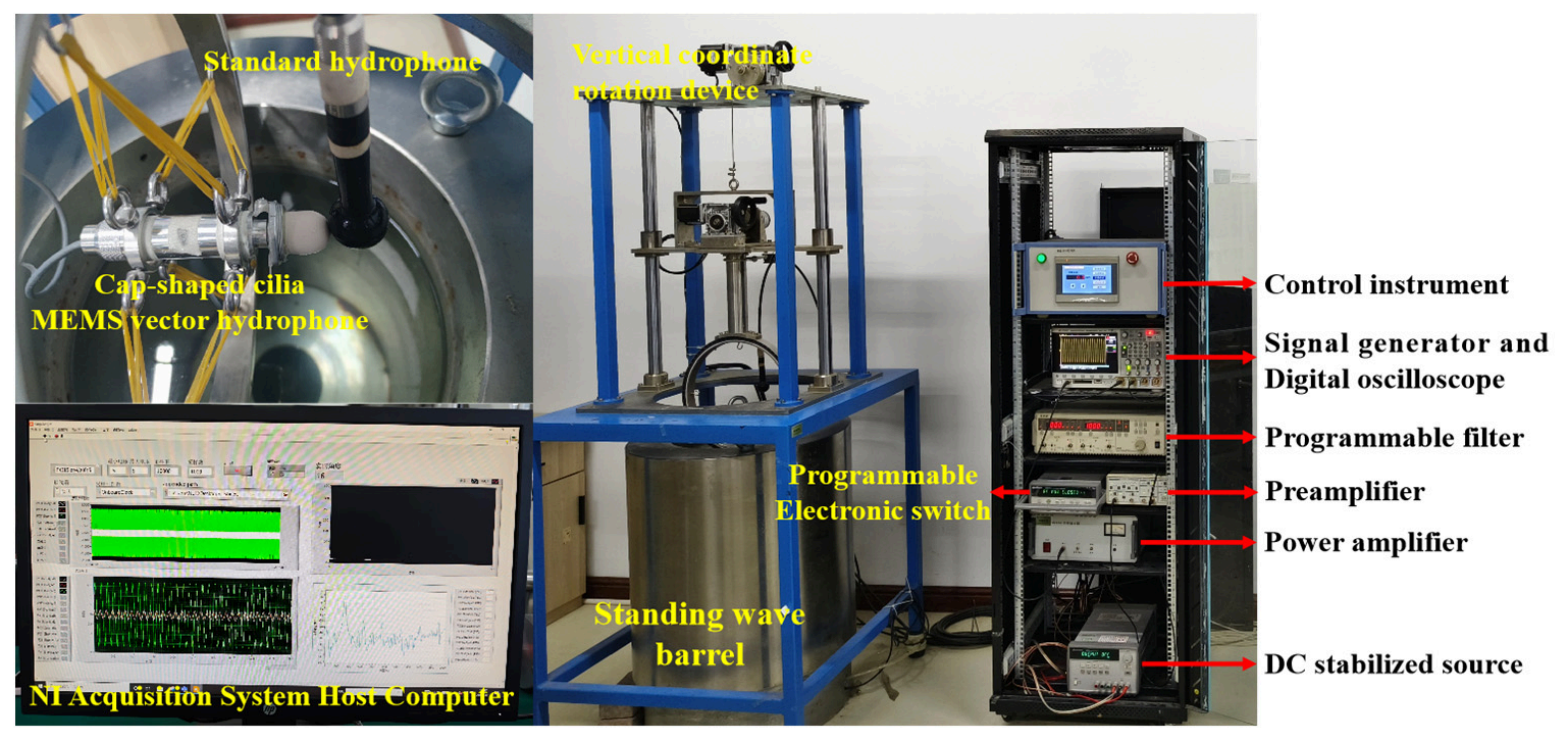
The test site of the low-frequency calibration system.

The test site of the low-frequency calibration system is shown in Figure 4. The procedure of the bearing history estimation experiment is described as follows:

1. The tested cap-shaped cilia MEMS vector hydrophone [5] is elastically suspended on a vertical rotation device.
2. The tested hydrophone is lowered by the controller to the same horizontal plane as the standard hydrophone, with a depth of approximately 15 cm.
3. The power supply device of the hydrophone is activated to transmit a sinusoidal pulse signal with an amplitude of 1 V and a frequency of 315 Hz. The MEMS vector hydrophone is rotated by 360° at a step interval of 2° via the vertical rotation device.
4. An external NI acquisition card is used for data sampling at a sampling rate of 10 kHz. After the experiment, the collected signals are analyzed to calculate the relative angle of the target.

**Figure 5:**
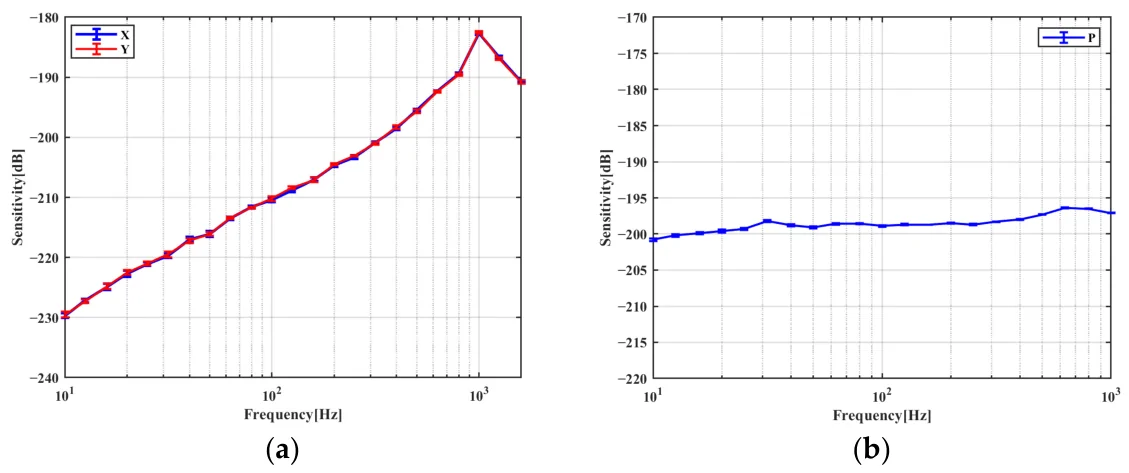
Sensitivity curve of sensor. (**a**) Vector channel; (**b**) scalar channel.

**Figure 6:**
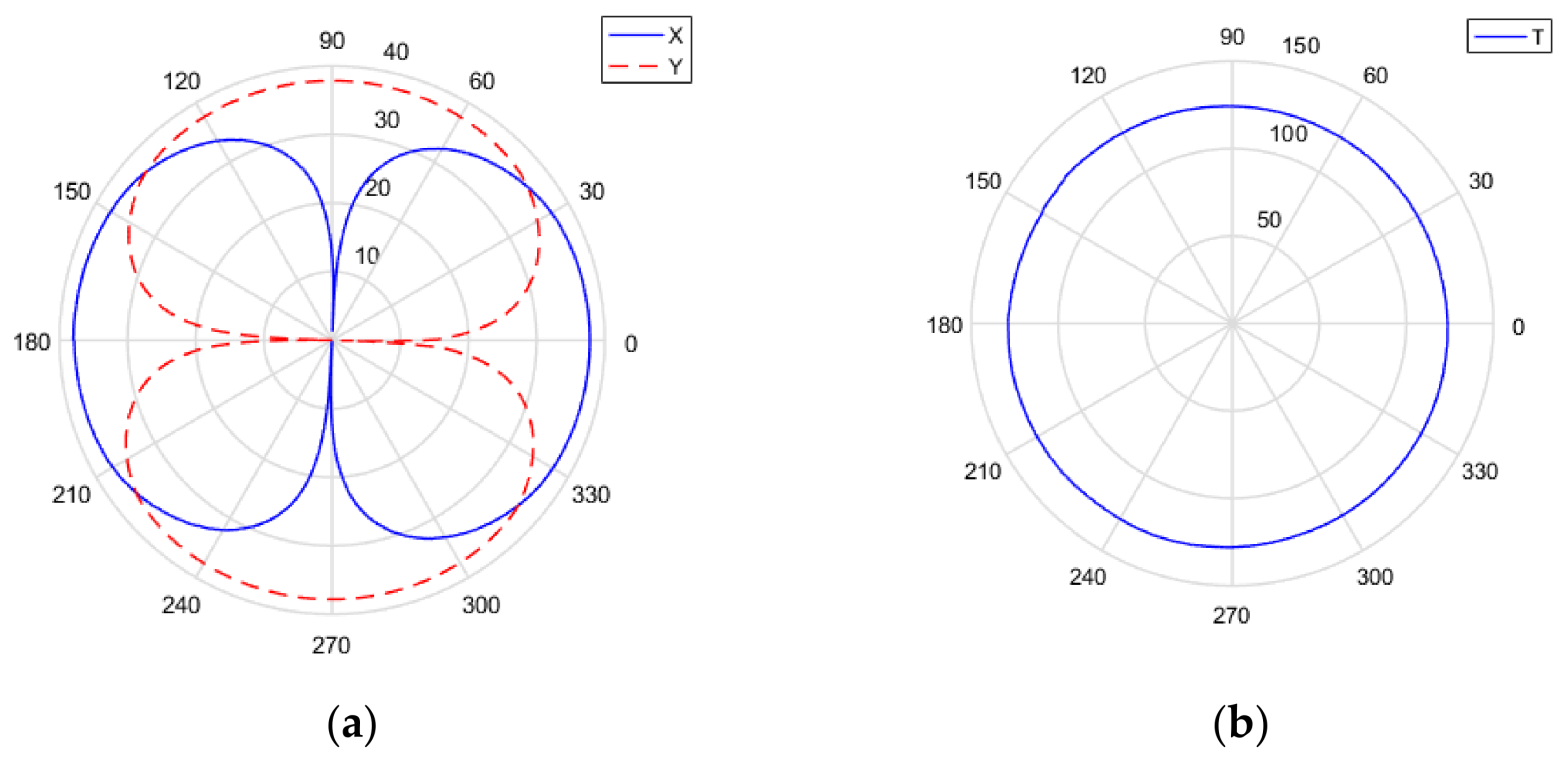
Directivity diagrams of sensor. (**a**) Vector channel; (**b**) scalar channel.

The sensitivity curve of the sensor is shown in Figure 5a,b. The curve is the result of repeated measurements. The measurement inaccuracy of the vector channel is within ±0.5 dB, and the measurement inaccuracy of the scalar channel is within ±0.2 dB. The vector channel sensitivity is −182.7 dB @1 kHz (0 dB@1 V/μPa), and the scalar channel sensitivity is −181.8 dB (0 dB@1 V/μPa). The directivity diagram measured at 315 Hz is shown in Figure 6a,b.

**Figure 7:**
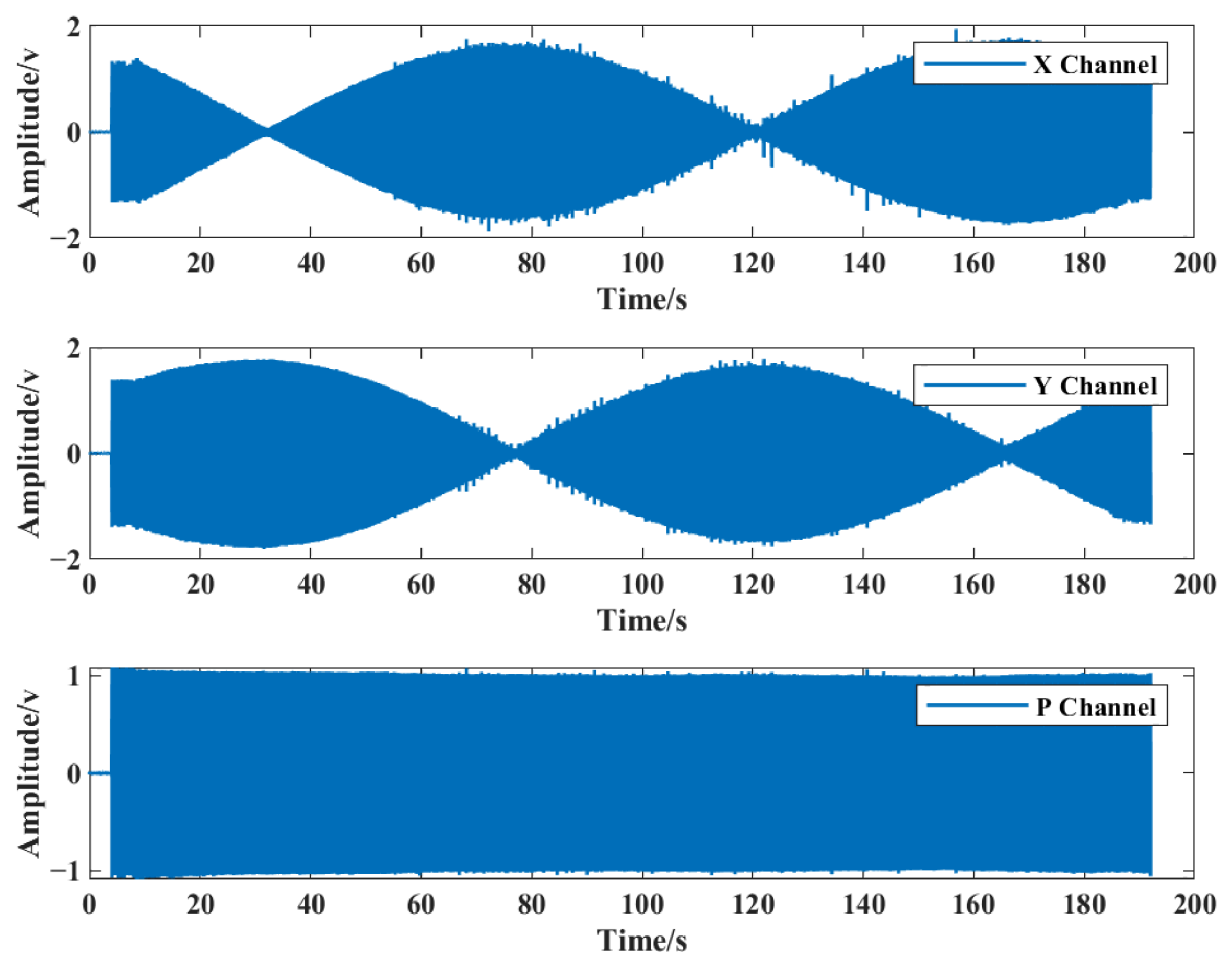
The time domain waveform of the sinusoidal signal.

**Figure 8:**
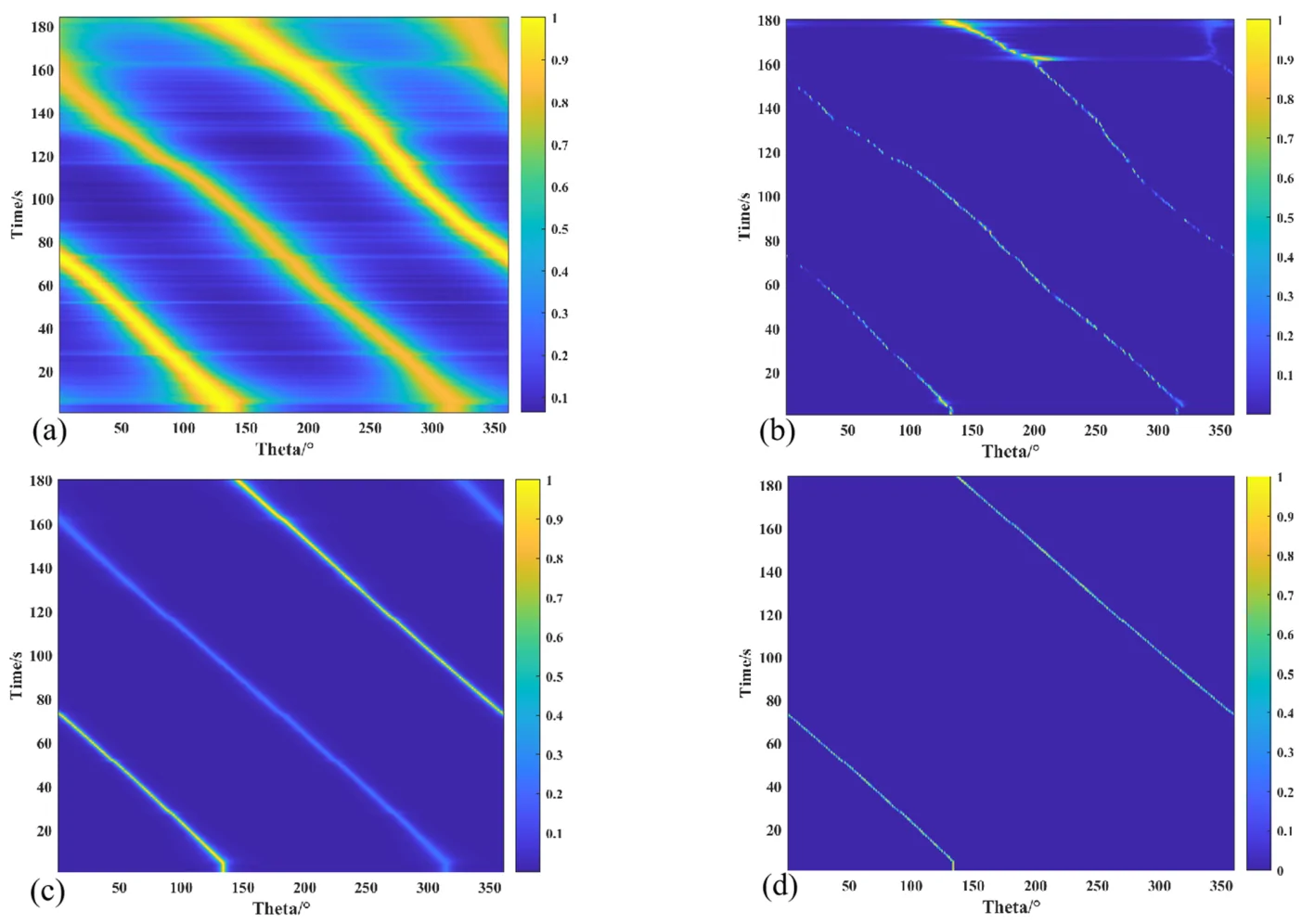
Bearing history estimation. (**a**) LV-MVDR; (**b**) Q-VV-MVDR; (**c**) Q-PVV-MVDR; (**d**) Q-PV-MVDR.

The time domain waveform of the sinusoidal signal received by the hydrophone is shown in Figure 7. The X and Y channels of the tested hydrophone maintain favorable orthogonality throughout the rotation period. The conventional MVDR algorithm and quaternion-based MVDR algorithms are adopted to analyze the target bearing trajectory. The bearing history estimation results are presented in Figure 8. It is observed that all the adopted algorithms can realize continuous 360° azimuth tracking within the dynamic test period of 180 s. The azimuth trajectory exhibits a double closed-loop characteristic of (314°→0°→360°→315°), which is consistent with the actual preset parameters. However, the signal intensity distribution of the LV-MVDR algorithm is not sufficiently concentrated and possesses the lowest resolution. The Q-VV-MVDR and Q-PVV-MVDR algorithms achieve higher resolution than the LV-MVDR algorithm, yet both suffer from the left–right ambiguity problem, where a spurious target appears at the mirror position. The emergence of such spurious targets may mislead the judgment of target azimuth and degrade the accuracy of direction-of-arrival estimation. In addition, the Q-PVV-MVDR algorithm provides better resolution than the Q-VV-MVDR algorithm. Among all the involved methods, the Q-PV-MVDR algorithm delivers the best overall performance. Its signal energy is well concentrated at the true target azimuth without generating any spurious targets, demonstrating excellent comprehensive performance.

### 4.2. Fixed-Point Bearing Estimation Experiment

**Figure 9:**
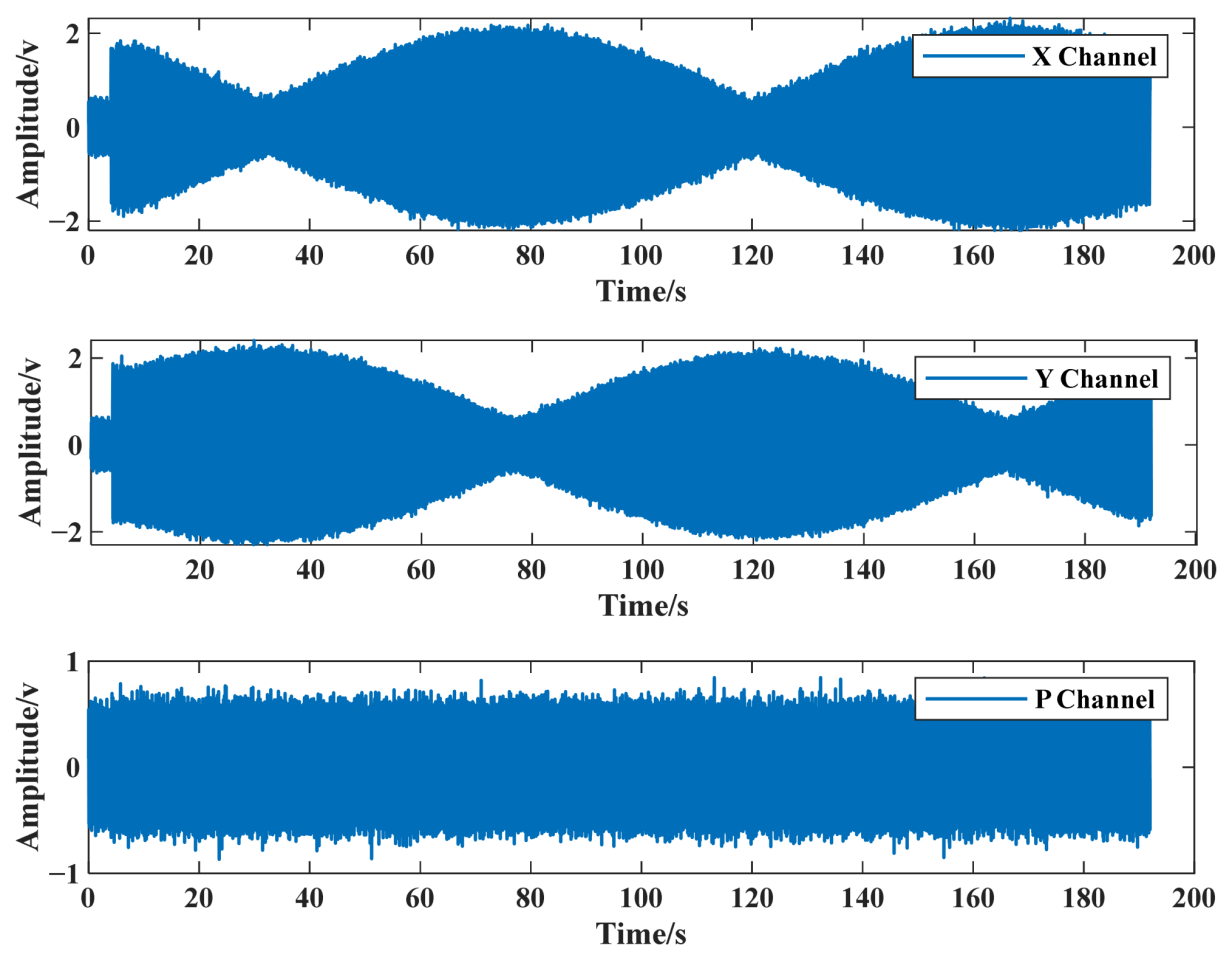
Time domain diagram of received signal after adding noise (SNR = −6 dB).

Sinusoidal signals are adopted as the transmitted signal to conduct the fixed-point bearing estimation test. The test is carried out within the range of 0° to 180° on the vertical rotation device with a test interval of 45°. Taking the 0° position as the starting point, the aforementioned algorithms are used to calculate the relative angular error. As can be seen from Figure 7, the original input signal has a relatively high SNR of 39 dB. To verify the effectiveness of the proposed method under low-SNR conditions, Gaussian white noise was added to the original signal. After adding noise, the SNR of the received signal decreased to −6 dB, as shown in Figure 9. Table 1 presents the azimuth estimation results of the rotating platform at different test angles under the noisy condition.

As illustrated in Table 1, all four algorithms achieve stable bearing estimation of the sinusoidal signal, with the proposed Q-PV-MVDR algorithm exhibiting the highest accuracy. In the fixed-point test, the bearing estimation error of the LV-MVDR algorithm ranges from 2° to 4°, with an average relative angle orientation error of 2.75°. The Q-VV-MVDR algorithm produces errors ranging from 0° to 4°, with an average relative angle orientation error of 2°. The error of the Q-PVV-MVDR algorithm ranges from 0° to 2°, with an average relative angle orientation error of 1°. In comparison, the Q-PV-MVDR algorithm limits the error to 0–1° and achieves the lowest average relative angle orientation error of only 0.5°. These results demonstrate that the proposed algorithm provides more accurate and stable bearing estimation under low-SNR conditions.

## 5. Conclusions

This paper proposes three quaternion-model-based MVDR algorithms for the direction-of-arrival estimation of a single MEMS vector hydrophone. Three quaternion data models are established for a single two-dimensional MEMS vector hydrophone, and the algorithms are improved according to the subspace decomposition principle to enhance the spectral peak resolution capability. The indoor experimental results show that the proposed three quaternion methods all achieve higher azimuth resolution and lower direction-finding error. Among them, Q-PV-MVDR can also effectively suppress the problem of port/starboard ambiguity. The fixed-point experimental results show that the average relative angle orientation error of the Q-PV-MVDR algorithm is only 0.5, and its positioning accuracy is significantly better than that of other comparison algorithms. The feasibility of the proposed quaternion MVDR algorithm is verified via an indoor low-frequency calibration system in this work. Nevertheless, field tests in lake waters and real marine environments have not yet been implemented. Future work will conduct in-depth investigations into complex issues such as environmental noise and platform constraints encountered in practical deployment.

## Figures and Tables

**Table 1:** Target azimuth estimation results of fixed-point test.

Rotating Device Angle	LV-MVDR		Q-VV-MVDR		Q-PVV-MVDR		Q-PV-MVDR	
	Estimated Value (°)	Error (°)	Estimated Value (°)	Error (°)	Estimated Value (°)	Error (°)	Estimated Value (°)	Error (°)
0°	2		2		2		2	
45°	−45	2	−45	2	−44	1	−44	1
90°	−94	4	−92	2	−89	0	−89	0
135°	−137	2	−137	0	−135	1	−134	0
180°	−179	3	−178	4	−178	2	−178	1
**Average error** (°)	2.75		2		1		0.5	

Note: 1. The error refers to the relative angular error. Taking the 0° position as the starting reference point, it is calculated by subtracting the previous fixed-point measurement position angle from the current estimated angle. 2. All estimated azimuth angles are expressed within the range of −179~180°. 3. The average relative angle orientation error is obtained by averaging the relative angular error data from 45° to 180°. 4. Test conditions: An indoor low-frequency test system, with a 250 Hz sinusoidal signal and a signal amplitude of 1 V. 5. SNR = −6 dB; since the vector channels (X channel, Y channel) are directional, the SNR is not unique at different times and angles. Therefore, the calculation of the SNR uses the scalar channel (P channel).

## Data Availability

Data is contained within the article.

## Abbreviations

The following abbreviations are used in this manuscript:

MVDR	Minimum variance distortionless response
DOA	Direction of arrival
MEMS	Micro-electro-mechanical system
REMS	Root mean square error
VSR	Vector signal reconstruction
SNR	Signal-to-noise ratio
MUSIC	Multiple signal classification
SVD	Singular value decomposition
LV-MVDR	Long-vector MVDR
Q-VV-MVDR	Quaternion vector–velocity MVDR
Q-PV-MVDR	Quaternion pressure–velocity MVDR
Q-PVV-MVDR	Quaternion pressure–vector–velocity MVDR

## References

[1] Liu Y., Jing B., Zhang G., Pei J., Jia L., Geng Y., Bai Z., Zhang J., Guo Z., Wang J. (2024). Design and Algorithm Integration of High-Precision Adaptive Underwater Detection System Based on MEMS Vector Hydrophone. Micromachines.

[2] Huang W., Sun S., Lu J., Xu Z., Xiu Z., Zhang H. (2026). A Multi-Task Learning Balanced Attention Convolutional Neural Network Model for Few-Shot Underwater Acoustic Target Recognition. Expert Syst. Appl..

[3] Wei M. (2012). Research of Localization Issues in Underwater Sensor Networks. Appl. Mech. Mater..

[4] Juan H., Kaiyu T. (2019). Simulation research of underwater passive direction finding technology. J. Acoust. Soc. Am..

[5] Zhu S., Zhang G., Shang Z., Yang X., Lv T., Liang X., Zhang X., Chen P., Zhang W. (2021). Design and realization of cap-shaped cilia MEMS vector hydrophone. Measurement.

[6] Zeng F., Han Y., Yang H., Yang D., Zheng F. (2025). Single Vector Hydrophone DOA Estimation: Leveraging Deep Learning with CNN-CBAM. Arch. Acoust..

[7] Liu Y., Yang Y., Huang Z., Fang S., Wang L. (2026). Design of a low-noise specific chip for the analog front end of MEMS piezoelectric hydrophone. Exp. Technol. Manag..

[8] Jia L., Zhang G., Liu Y., Bai Z., Geng Y., Wu Y., Zhang J., Zhang W. (2025). Sonar buoy active detection and localization for underwater targets using high-level sound sources and MEMS hydrophone. Measurement.

[9] Sun G., Yang D., Shi S. (2003). Spatial correlation coefficients of acoustic pressure and particle velocity based on vector hydrophone. Acta Acust..

[10] Shi L., Zhou N., Wu J., Shi M., Shi Y., Lei C., Mao H. (2025). An All-in-One Testing Chip for the Simultaneous Measurement of Multiple Thermoelectric Parameters in Doped Polysilicon. Micromachines.

[11] Ahangari M., Zhou M., Luo H. (2025). Review of Layered Transition Metal Oxide Materials for Cathodes in Sodium-Ion Batteries. Micromachines.

[12] Hsu J.-C., Liao K.-L. (2025). Driving Rotational Circulation in a Microfluidic Chamber Using Dual Focused Surface-Acoustic-Wave Beams. Micromachines.

[13] Ganguly S., Margel S. (2025). Magnetic Polymeric Conduits in Biomedical Applications. Micromachines.

[14] Zhang X., Zhang G., Shang Z., Zhu S., Chen P., Wang R., Zhang W. (2021). Research on DOA Estimation Based on Acoustic Energy Flux Detection Using a Single MEMS Vector Hydrophone. Micromachines.

[15] Guo J. (2024). Sparse Direction of Arrival Estimation Method Based on Vector Signal Reconstruction with a Single Vector Sensor. arXiv.

[16] Geng Y., Dai W., Zhang G., Wu L., Zhang J., Jia L., Wang J., Zhao H., Bai Z., Zhou Z. (2025). Frequency-Domain Deconvolution Adaptive Noise Cancellation DOA Estimation Algorithm Based on Matched Filtering. IEEE Trans. Instrum. Meas..

[17] Zhang B., Liu Y., Gao T., Yin J., Guan Z., Zhang D., Zeng L. (2025). Automatic Extraction and Compensation of P-Bit Device Variations in Large Array Utilizing Boltzmann Machine Training. Micromachines.

[18] Hawkes M., Nehorai A. (1998). Acoustic vector-sensor beamforming and Capon direction estimation. IEEE Trans. Signal Process..

[19] Cao J., Liu J., Wang J., Lai X. (2017). Acoustic vector sensor: Reviews and future perspectives. IET Signal Process..

[20] Wang F., Wang S., Wu Y. 2-D DOA Estimation in the Presence of Gaussian Noise with Quaternion. Proceedings of the 2005 IEEE International Symposium on Microwave, Antenna, Propagation and EMC Technologies for Wireless Communications.

[21] Gong X., Liu Z., Xu Y. (2009). Quad-Quaternion MUSIC for DOA Estimation Using Electromagnetic Vector Sensors. Eurasip J. Adv. Signal Process..

[22] Meng T., Wu M., Yuan N. (2017). DOA Estimation of Noncircular Signals Using Quaternions. Int. J. Antennas Propag..

[23] Wang G., Wang X., Wang L., Wang X. (2020). Research on Ambiguity Resolution Algorithm by Quaternion Based on Acoustic Vector Sensor. J. Sens..

[24] Jamshidpour S., Sakhaei S.M. (2022). Polynomial Rooting-Based DOA Estimation Algorithm for Vector-Sensor Arrays Using Quaternions. Multidimens. Syst. Signal Process..

[25] Lou Y., Qu X., Zhang R., Zhou Z., Cheng J., Yuen C. (2025). Performance Analysis of Quaternion-MUSIC: Unification, Simplification, and Evaluation. Signal Process..

[26] Busacca F., Galluccio L., Palazzo S., Panebianco A. (2024). A comparative analysis of predictive channel models for real shallow water environments. Comput. Netw..

[27] Ben Taher R., Schmüdgen K., Zerouali E.H. (2026). The Quaternionic Moment Problem. J. Algebra.

[28] Geng Y., Zhang G., Zhang J., Yin C., Zhang F., Hao J., Wang M., Bai Z., Yang Y., Zhang W. (2026). Research on Azimuth Estimation Algorithm for Explosion Sound Signals Based on Single-Vector Hydrophone. Def. Technol..

[29] Manolakis D.G., Ingle V.K., Kogon S.M. (2005). Statistical and Adaptive Signal Processing: Spectral Estimation, Signal Modeling, Adaptive Filtering and Array Processing.

[30] Muni B.K., Delwar T.S., Panigrahi T., Pradhan P.K., Ra I.-H., Kim H.-J., Kaur M., Hosen A.S.M.S., Ryu J.-Y. (2026). A Robust Direction of Arrival Estimation Method Based on the Chaotic MUSIC Algorithm. Sci. Rep..

[31] Nguyen T.K., Nguyen N.D., Nguyen H.Q., Nguyen K.T.V. (2025). Signal Enhancement and Interference Reduction with Minimum-Variance Distortionless Response Algorithm Using MATLAB and GNU Radio Simulations. Eng. Proc..

[32] Guo S.L., Tang R.C., Peng L.H., Ji X.P. (2016). Matched Field Localization Based on CS-MUSIC Algorithm. J. Ocean Univ. China.

